# Properties of Luffa Fiber Reinforced PHBV Biodegradable Composites

**DOI:** 10.3390/polym11111765

**Published:** 2019-10-27

**Authors:** Yong Guo, Li Wang, Yuxia Chen, Panpan Luo, Tong Chen

**Affiliations:** College of Forest and Garden, Anhui Agricultural University, Hefei 230036, China; guoyong@ahau.edu.cn (Y.G.); 18356092360@163.com (L.W.); 15005170106@163.com (P.L.); 18356092289@163.com (T.C.)

**Keywords:** luffa fiber (LF), NaOH-H_2_O_2_, composite, water absorptivity, bending property

## Abstract

In this study, composites of poly (hydroxybutyrate-co-valerate) (PHBV) with untreated luffa fibers (ULF) and NaOH-H_2_O_2_ treated luffa fibers (TLF) were prepared by hot press forming. The properties of luffa fibers (LFs) and composites were characterized by scanning electron microscopy (SEM), Fourier transform infrared spectroscopy (FTIR), and other analysis methods. Results showed that pre-treatment effectively removed pectin, hemicellulose, and lignin, thus reducing the moisture absorptivity of LFs. The flexural strength of TLF/PHBV was higher than that of ULF/PHBV. With 60% LF content, the flexural strengths of ULF/PHBV and TLF/PHBV reached 75.23 MPa and 90.73 MPa, respectively, 219.7% and 285.6% more than that of pure PHBV. Water absorptivities of composites increased with increase in LF content. Water absorptivity of TLF/PHBV was lower than that of ULF/PHBV. The flexural strengths of composites decreased after immersion in water at room temperature. Meanwhile, flexural strength of TLF/PHBV was lower than that of ULF/PHBV. Pretreatment of LFs effectively improved the bonding between fibers and PHBV, resulting in enhanced and thus improved the moisture resistance of composites.

## 1. Introduction

Luffa is an annual dimbing plant belonging to the Cucurbitaceae family, which is widely grown in hot and humid regions such as China, Japan, India, as well as Central and South America, and other countries [1,2]. Luffa fiber (LF) is a bundle of natural vascular tissue with fibrous reticular structure obtained from ripe luffa fruits. The portion remaining after removing the skin and seeds is also known as luffa sponge or plant sponge [3]. Natural LFs are widely used for preparing cleaning products, bedding products, insoles, toys, etc. However, the manufacture of these products often involves a large number of winding corners and generates waste. In order to improve the utility and increase the value of these wasted LFs, further research is necessary. With continuous developments in modern science and technology, LF, a green renewable resource with unique light porous three-dimensional network structure, good permeability, adsorptivity, and antimicrobial properties, is also completely degradable. LFs are edible and used in traditional medicine, in addition to their applications in biotechnology [4], clinical medicine [5,6], mattress materials [7], etc. Research on LF reinforced polymer composites has also been carried out vigorously [8,9], due to their excellent comprehensive properties, making them good reinforcing phases for composite materials [10,11,12,13].

Fiber reinforced polymer composites have been developed since the beginning of 20th century. They are widely used in military and civil fields, due to their high specific strength and modulus [14]. Common polymers such as polypropylene (PP), polyethylene (PE), and polyvinyl chloride (PVC) are commonly synthesized from non-renewable resources, like petroleum and natural gas. Most of them are difficult to degrade and the degradation products are generally harmful. In recent years, due to shortage of petroleum resources and concerns of environmental pollution, there is great demand for developing new green composite materials, which have excellent performance, are economical, environmentally friendly, and completely biodegradable. Natural fibers are used as reinforcing materials to prepare fully degradable composites. On one hand, the reuse of agricultural and forest wastes can reduce the pressure on resources and environment. On the other hand, improvement in mechanical properties of biodegradable plastics can be achieved along with reduction in cost. Therefore, natural fiber reinforced biodegradable plastics have attracted wide attention.

Poly(hydroxylalkanes) (PHAs), produced from starch by controlled bacterial fermentation, are one of the most widely used and promising biodegradable polymers. Common PHAs include poly(hydroxybutyrate) (PHB), poly(hydroxybutyrate-*co*-valerate) (PHBV), and poly(3-hydroxybutyrate-*co*-3-hydroxy hexanoate) (PHBH). Among them, PHBV has excellent mechanical properties. When the molar content of the poly-3-hydroxyvalerate (HV) segment is 3% to 8%, the thermal and mechanical properties are similar to those of traditional thermoplastics such as polyethylene (PE). PHBV also has significant biodegradability, biocompatibility, and optical activity and finds applications in many fields [15,16]. However, some inherent properties of biodegradable plastics have limited their further utilization such as high cost, high brittleness, easy decomposition, and low impact resistance. Therefore, researchers have made attempts to improve the performance of PHBV by different means in recent years [17,18,19]. There have been gradual developments in natural fiber reinforced PHBV matrix. S Peterson et al. studied the mechanical properties and biodegradability of wood fiber/PHBV composites, prepared by hot pressing process [20]. S Luo et al. studied the interfacial bonding properties of pineapple leaf fiber and agave fiber reinforced PHBV composites [21,22]. Singh compared the properties of bamboo fiber/PHBV and pure PHBV and found that the tensile modulus and heat deflection temperature of composite at 40 wt.% fiber increased by 175% and 9 °C, respectively [23].

Natural fibers have many advantages compared with synthetic fibers. However, they also have some disadvantages, such as high water absorptivity, low thermal stability, and large variations in properties [24]. Hence, chemical modification of fibers is essential to improve their properties. In addition, improvement of interfacial compatibility between hydrophilic polar plant fibers and hydrophobic nonpolar polymer matrix has always been one of the biggest problems in the preparation of composite materials. Methods to improve the interfacial properties include alkali treatment of plant fibers [25], modification of plant fibers using coupling agent [26], and plastic modification [27].

LF contains large amounts of hemicellulose, cellulose, and lignin. Chen et al. showed that LF, a natural fiber, had higher moisture absorptivity compared to jute because of its porous structure and presence of surface grooves in LF bundles [28]. Use of fiber as a reinforcing material in composites, causes them to expand after water absorption. This leads to formation of micro-cracks in the composites, thus reducing their mechanical properties [29]. In order to make better use of LF and reduce its moisture absorptivity, chemical modification is generally carried out. This includes alkali treatment to effectively remove hemicellulose and lignin [30]. Chen et al. used three different chemical methods for treatment of LFs. Results showed that 5% NaOH-5% H_2_O_2_ could significantly reduce water absorptivity and effectively remove hemicellulose and lignin [29].

In order to enhance interfacial bonding in the composites and reduce water absorptivity of the composites, LFs were treated with 5% NaOH-5% H_2_O_2_ solution. Then untreated luffa fiber/PHBV (ULF/PHBV) and treated luffa fiber/PHBV (TLF/PHBV) biodegradable composites were prepared by hot pressing. The thermal degradation, crystallinity, water absorptivity, and bending properties of the composites were studied. In addition, the bending properties of the composites before and after immersion in water were tested.

## 2. Materials

Bamboo powder (Anhui, China) was grinded and prepared into powder in laboratory and was passed through 80 mesh sieves. The ripe LFs (Jiangxi, China) were powdered using a plant grinder and passed through 60 mesh sieves. Commercially available PHBV (Guangzhou, China) was used as the matrix.

### 2.1. Chemical Treatment of the Fibers

LFs were pre-dried in an air blast drying oven at 50 °C for 8 hours to remove water. A mixed solution containing 5% NaOH and 5% H_2_O_2_ in a volume ratio of 1:1 was taken in a flask and placed in a water bath at 85 °C. The dried LFs were immersed in the above solution for one hour while stirring mechanically. The solid–liquid ratio was 1:10. The TLFs were fully washed with distilled water. After two days of air-drying at room temperature, the LFs were dried in an air blast drying oven at 80 °C.

### 2.2. Fabrication of Composites

**Figure 1:**
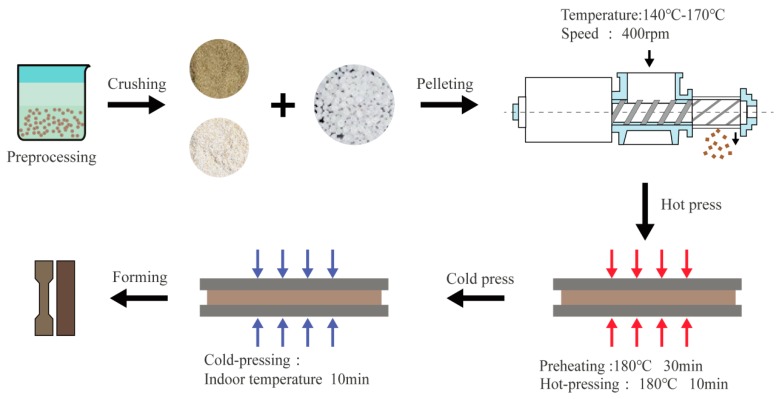
Schematic illustration of the fabrication of composites.

LFs were placed in a humidity chamber with relative humidity (RH) of 65% for 24 h at 21 °C and then dried for 24 h at 105 °C in a blast drying box to reduce the moisture content to less than 3%. Based on varying LF mass fractions (0%, 30%, 40%, 50%, 60%, 70%, and 80%), LF and PHBV were mixed in a granulator. The temperature of each granulation zone was maintained between 140–170 °C with main engine speed of 400 rpm. The obtained granules were preheated by hot pressing and then pressurized to 7 MPa for 10 min. The sheet was then cold pressed at room temperature for 10 min at 7 MPa. The steps for preparation are shown in Figure 1.

### 2.3. Characterization of Fibers and Composites

The contents of hemicellulose, *α*-cellulose, and lignin contents in LFs were determined according to GB/T 2677.10-1995, GB/T 744-1989, and GB/T 2677.8-1994 standards, respectively. Hemicellulose content was determined by subtracting the *α*-cellulose and lignin contents from the combined weight of the three components. The final results were the average of three readings.

The thermal degradation properties of composites were tested on a thermal analyzer (TGA209F3, Netzsch, Bavaria, Germany). Each sample (5–10 mg) was taken in a platinum crucible and tested in dynamic nitrogen atmosphere at a heating rate of 10 °C /min from 25 to 700 °C.

The dried LF sample and potassium bromide (KBr; 1 mg powder/100 mg KBr) were pressed together into pellets for FTIR analysis (Bruker, Billerica, MA, USA). Each sample was scanned 32 times at 4 cm^−1^ resolutions.

The morphology of LF and the cross-sections of composites were observed by field emission scanning electron microscopy (FESEM, Hitachi Co., Ltd., S-4800, Tokyo, Japan). The fractured surfaces of the samples were coated with a thin layer of gold before analysis and the samples were analyzed at an accelerating voltage of 3.0 kV.

A Universal Mechanical Testing Machine (Shimadzu Corporation, Shimadzu AG-X Plus, Kyoto, Japan) was used to test the flexural strengths and moduli of composites according to ASTM D790 standard. A 1.0 kN load cell was employed with crosshead speed of 2 mm/min. The test was repeated five times for each ratio.

Water absorptivities were tested according to ASTM D570-98 standard. Three specimens of each sample were dried in the oven at 50 ± 3 °C for 24 h and immediately weighed to an accuracy of 0.001 g. The specimens were then immersed in distilled water at 23 ± 1 °C for 24 h. Subsequently, the test specimens were carefully dried using a dry cloth and immediately weighed to an accuracy of 0.001 g. The water absorption was calculated using Equation (1):

(1) $$W\%=\frac{\left(W_{1}-W_{0}\right)}{W_{0}}\times 100\%$$

Here, *W* (%) is the water absorption percentage; *W*_1_ is the weight of the wet sample after 24 h, g; and *W*_0_ is the initial weight of the sample, g.

## 3. Results and Discussion

### 3.1. Properties of LF before and after Treatment

Table 1 shows the changes in chemical compositions and moisture absorptivities of LFs, before and after treatment [29]. Compared to ULFs, the *α*-cellulose content in TLFs was increased, while that of hemicellulose and lignin decreased. This was accompanied by a decrease in hygroscopicity. The ULF had a high moisture absorption rate, because it contained hydrophilic pectin and nitrogen-containing groups on its surface. In addition, ULFs had higher hemicellulose and lignin contents, and more number of hydrophilic –OH groups. Treatment with NaOH–H_2_O_2_ helped to remove wax, oil, hemicellulose, and lignin from LFs. It also led to reduction in the number and types of hydrophilic groups [29]. Hence, the hemicellulose and lignin contents and moisture absorptivity of LFs were reduced after treatment.

**Figure 2:**
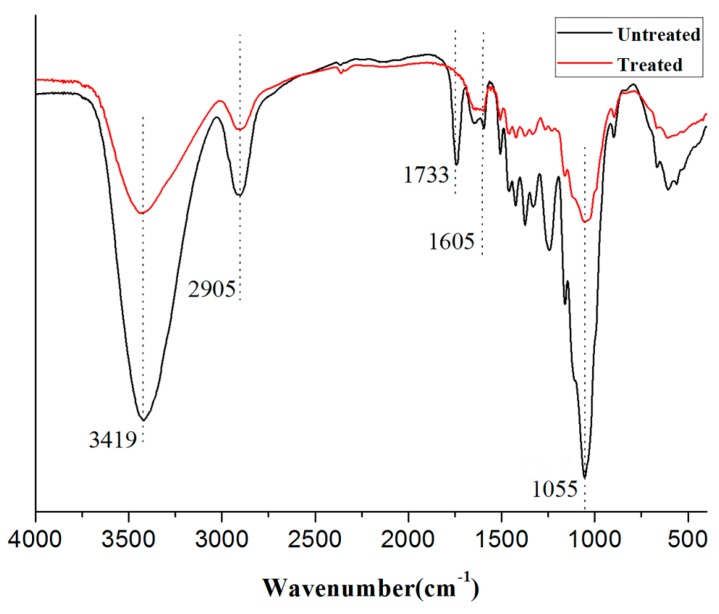
FTIR spectra of LF before and after treatment [29].

Figure 2 shows the FTIR spectra of LF before and after treatment with NaOH–H_2_O_2_. The peak at 3419 cm^−1^ could be assigned to the hydroxyl groups of cellulose, hemicellulose, and monosaccharide molecules [31]. After treatment with NaOH–H_2_O_2_, the peak for the stretching vibration of –OH was weakened, indicating that alkali treatment caused a reduction in the number of hydroxyl groups on the fiber surface [32]. The peak at 2905 cm^−1^ was due to C–H stretching vibrations of CH and CH_2_ groups in cellulose and hemicellulose components [33]. Peak near 1055 cm^−1^ was due to the C–O bonds in cellulose and hemicellulose [34]. The peak at 1733 cm^−1^ could be ascribed to the C=O stretching vibrations in lignin and hemicellulose [35]. The peak centered at 1605 cm^−1^ was assigned to the C=C aromatic conjugated C–C bond tensile vibration peak, characteristic of lignin [36]. The intensities of all four peaks decreased significantly after treatment, indicating that hemicellulose and lignin were effectively removed from TLF [29].

**Figure 3:**
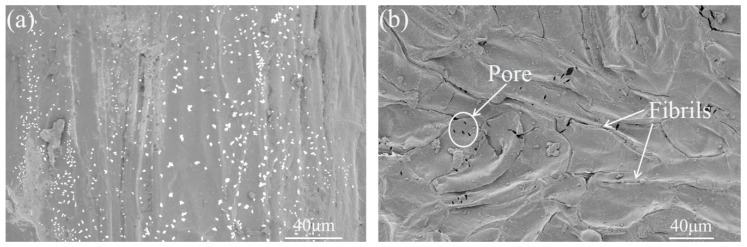
SEM images of LF, (**a**) untreated LF (ULF), and (**b**) treated LF (TLF).

Figure 3a,b are the SEM images of untreated and NaOH–H_2_O_2_ treated LF bundles. Results showed that the surface of ULF had some pores, groves, and microcracks [29]. The surface of the fiber bundle showed a large number of white uniformly sized spot-like impurities of pectin and wax. After the NaOH–H_2_O_2_ treatment, the LF surface was rougher than that of ULF. The grooves became denser along the axis, and the surface area of LFs was increased. This could be explained by the fact that the alkali treatment promoted the removal of hemicellulose, wax, and lignin on the surface of LF [37]. Due to the action of H_2_O_2_, the conjugated carbonyl groups of lignin side chains underwent oxidation [38], which further reduced the lignin content. In addition, alkali and hydrogen peroxide reduced the number of hydroxyl groups on the surface of LF, thereby reducing the polarity of the fiber. The increase in roughness of the fiber surface and decrease in the number of hydroxyl groups promoted the mechanical interlocking and collective combination between the fibers and the resin.

### 3.2. Properties of Composite Materials

#### 3.2.1. FTIR Spectra of Composites

**Figure 4:**
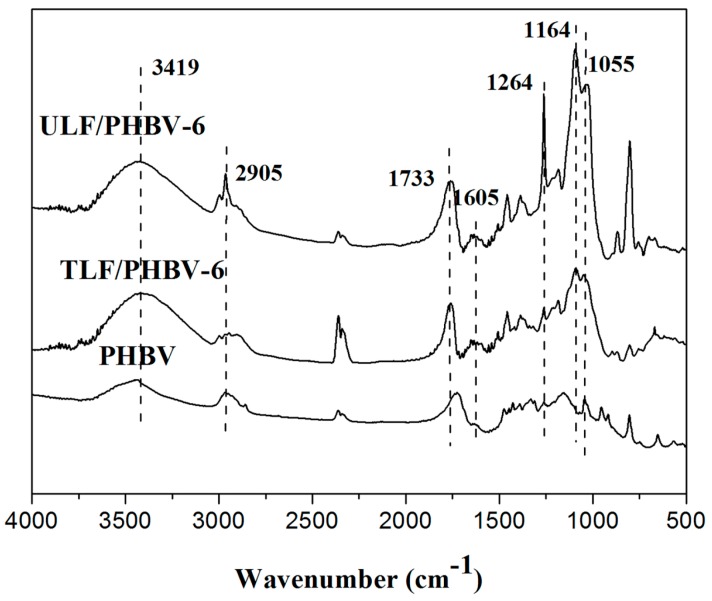
FTIR spectra of composites.

Figure 4 shows the FTIR spectra of PHBV, ULF/PHBV, and TLF/PHBV. The stretching vibration peak at 3419 cm^−1^ for –OH in pure PHBV might be caused by the absorption of water molecules and the vibration of the associated –OH. After the addition of LF, this peak increased significantly. It might be derived from cellulose, hemicellulose, polysaccharides, and mono-saccharide molecules –OH. It can be seen that the composites treated by NaOH and H_2_O_2_ had a weaker peak for –OH stretching vibration at 3419 cm^−1^, which was caused by the reduction in the number of hydroxyl groups on the surface of the LF treated by NaOH and H_2_O_2_. At the same time, the peaks of ULF/PHBV at 1605 cm^−1^, 1733 cm^−1^, and 2905 cm^−1^ were higher than those of TLF/PHBV and pure PHBV. This is because the untreated LF had a high content of hemicellulose and lignin, and therefore had a large number of chemical bonds such as C–H, C=O, and C–C.

The peak at 1164 cm^−1^ was for the C–O–C asymmetric stretching vibration, which was an absorption peak for an ether bond or an acetal bond. These functional groups were the chemical basis for the polarity of the surface of LFs. In contrast, the functional groups of PHBV were mostly non-polar, which was also the root cause of poor bonding strength between PHBV and LFs. Therefore, having a small polarity on the LF surface was advantageous to increase the interface strength between. The peak of ULF/PHBV was much larger than that of TLF/PHBV. Therefore, the interface bonding strength of ULF and PHBV was lower than that of TLF. This was also reflected and confirmed by the following mechanical properties and SEM images.

#### 3.2.2. Water Absorptivity

**Figure 5:**
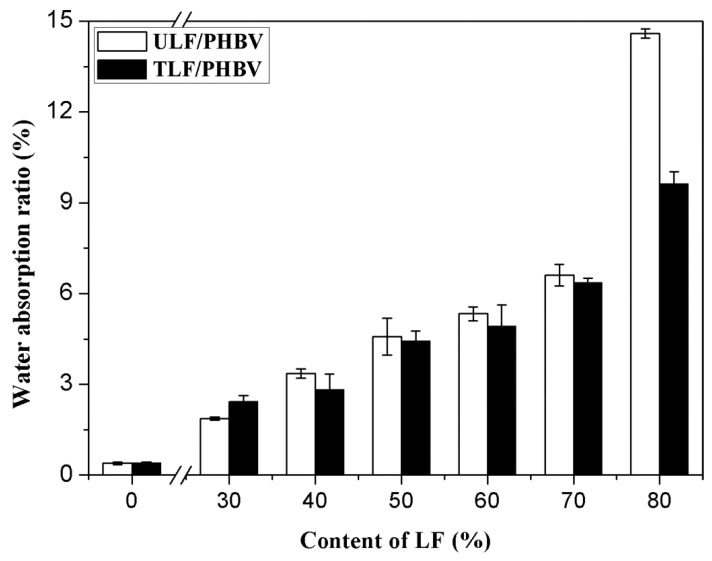
Water absorptivity of the composites with different LF contents.

Figure 5 shows the effect of the LF content on the water absorptivity of composites. The water content of pure PHBV was 0.39%. With an increase in LF content, the water absorptivity of the composites increased continuously and all of them exceeded that of pure PHBV. The moisture contents of ULF/PHBV and TLF/PHBV reached maximum values of 14.98% and 10.49%, respectively, when the LF content was 80%. When the LF content was 60%, the water absorption rate of TLF/PHBV was 4.47%, which was 7.07% lower than that of ULF/PHBV.

The water absorptivities of the composites were not only dependent on the nature of fibers, but also on the binding at the interfaces of plant fibers and PHBV [39]. Pure PHBV had good hydrophobicity, whereas LF, as a natural plant fiber, had good hygroscopicity, which affected the water absorptivities of composites. With an increase in LF content of the composite, PHBV was unable to completely encapsulate the plant fibers. Their combination was poor, due to which there were more channels for water to enter the composites. In addition, the contact area between fibers and water was also large, which led to an increase in water absorptivities of composites. After the NaOH–H_2_O_2_ treatment, the area of contact between LF and PHBV increased, the interfacial bonding strengthened, and the voids in composites decreased. This made the entry of moisture difficult, and reduced the water absorptivities of the composites. After alkali treatment, hemicellulose was removed due to reaction of –OH groups, which also resulted in decrease in number of surface –OH groups of LF and consequently reduction in water absorptivity [32]. When the LF content was 30%, the water absorption rate of TLF/PHBV was slightly higher than that of ULF/PHBV. This could be due to the formation of many small bubbles as a consequence of uneven mixing during the preparation, which hindered the penetration of moisture.

#### 3.2.3. Thermogravimetry

**Figure 6:**
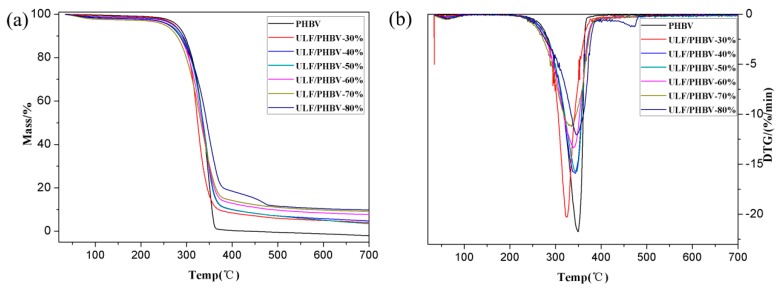
TGA and DTG curves of ULF/PHBV composites: (**a**) TGA and (**b**) DTG.

**Figure 7:**
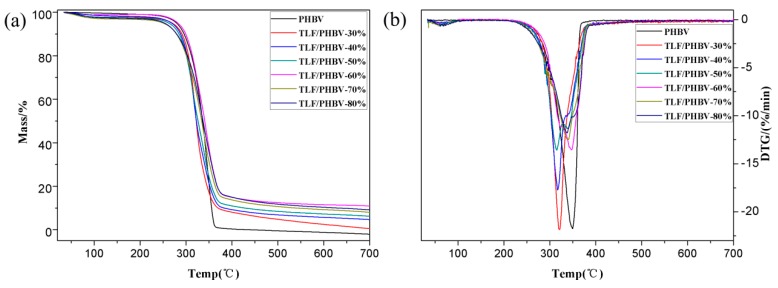
TG and DTG curves of TLF/PHBV composites: (**a**) TG and (**b**) DTG.

Figure 6 and Figure 7 show the TG and differential thermal gravity (DTG) curves of PHBV, ULF/PHBV, and TLF/PHBV and the corresponding data are list in Table 2. TG curves of PHBV, ULF/PHBV, and TLF/PHBV composites in Figure 6 showed single-stage degradation patterns with typical “Z” shape. The initial degradation temperature (*T*_initial_) of pure PHBV was 313.9 °C. After adding LF, the *T*_initial_ values of ULF/PHBV and TLF/PHBV were significantly decreased. The LF content was 60%, the *T*_initial_ values of the composites were 296.0 °C and 300.8 °C, a reduction of 17.9 °C and 13.1 °C, respectively, compared with pure PHBV. This was mainly due to the fact that the composite had a large amount of LF exposed to the surface. Besides, the heat resistance of hemicellulose was worse than that of PHBV, which resulted in early decomposition of exposed fibers [40]. The hemicellulose dissolved partially after NaOH–H_2_O_2_ treatment, which increased the *T*_initial_ of TLF/PHBV.

PHBV was completely degraded (more than 99%) at 491.4 degree °C, whereas the masses of ULF/PHBV and TLF/PHBV remained unchanged at about 500 °C. The residual amounts of composites were higher than that of pure PHBV, mainly due to the carbides and ash produced during LF combustion. The weight loss rate of PHBV sample was 97.23% in the peak degradation temperature range. However, the weight loss rates of ULF/PHBV and TLF/PHBV were 85.00% and 83.56%, respectively, when the LF content was 60%. In addition, the weight loss rate of TLF/PHBV in the peak degradation temperature range was lower than that of ULF/PHBV. This indicated that the thermal degradation rate of PHBV could be reduced by adding LF, while the thermal degradation rate of the composite could be further reduced by treatment with NaOH–H_2_O_2_. The ULF/PHBV and TLF/PHBV samples with 80 wt. % fiber clearly presented a second decomposition stage. This may be because a large number of LFs agglomerated in the composite, causing the composite to burn a large amount of underburned char. The increased heating resulted in carbon degradation.

In general, LFs greatly improved the char formation of the system. The increase of LFs slowed down the rate of thermal decomposition of bamboo–plastic composites at high temperatures and prolonged the time of thermal decomposition.

#### 3.2.4. Mechanical Property

**Figure 8:**
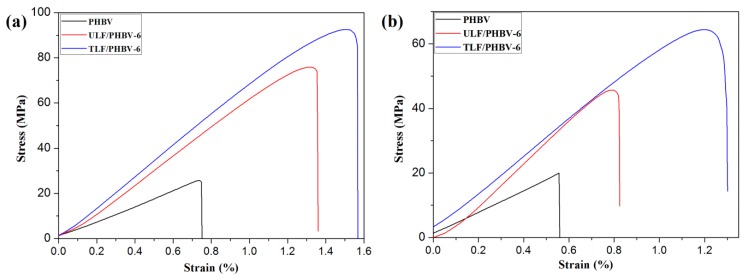
Stress–strain responses for composites: (**a**) non-immersion and (**b**) after immersion.

Stress–strain responses for composites before immersion and after immersion are shown in Figure 8. With or without water absorption, TLF/PHBV-6 exhibited maximum ultimate flexural stress and flexural modulus, followed by ULF/PHBV, and pure PHBV. After immersion, the maximum bending strengths of PHBV, ULF/PHBV, and TLF/PHBV were reduced, and the decrease in bending strength of pure PHBV was the smallest.

**Figure 9:**
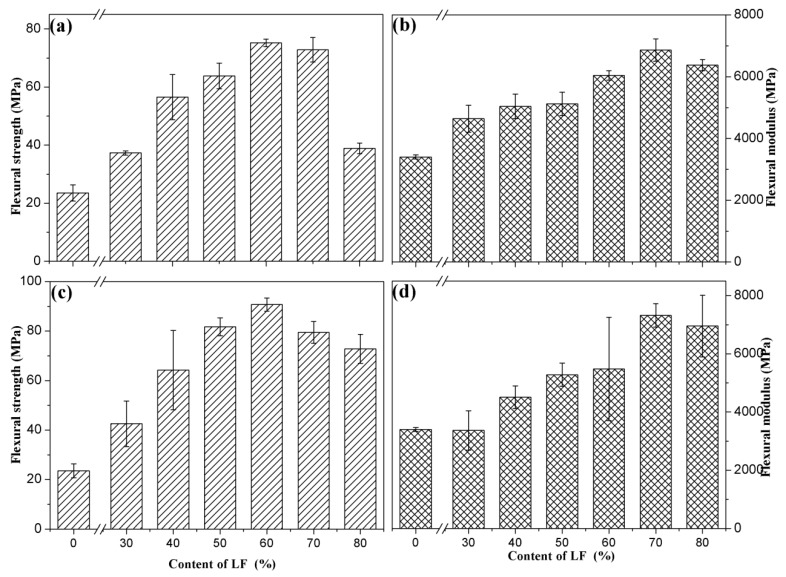
Flexural strengths and moduli of composites: (**a**,**b**) ULF/PHBV and (**c,d**) TLF/PHBV.

Figure 9 shows the effects of LF content on the flexural properties of ULF/PHBV and TLF/PHBV composites. Pure PHBV had flexural strength and modulus of 23.53 MPa and 3401.41 MPa, respectively. It can be seen from Figure 9a,b that the flexural strengths and moduli of ULF/PHBV and TLF/PHBV composites increased at first and then decreased with an increase in LF content. When the LF content was 60%, the flexural strengths of ULF/PHBV and TLF/PHBV composites reached the maximum values of 75.23 MPa and 90.73 MPa, respectively. This corresponded to an increase of 219.7% and 285.6%, respectively, compared with pure PHBV. When the LF content was less than 60%, it was uniformly dispersed in PHBV matrix and effectively wrapped by it. As the LF content increased, the fibers were evenly distributed in the PHBV matrix and were intertwined forming an interlocked network structure. This structure was able to withstand an external load, indicating that LF could enhance the mechanical properties of the composite. When the LF content continued to increase to more than 60%, the PHBV matrix could not uniformly wrap the fibers and hence a large number of fibers were exposed. The extent of interfacial bonding and bonding strength between the matrix and the fibers decreased. In addition, due to high fiber content, aggregation of fibers occurred readily during the mixing process, resulting in concentration of stress in the composite materials [41]. When the LF content was 70%, the flexural moduli of ULF/PHBV and TLF/PHBV composites reached maximum values of 6865.3 MPa and 7325.3 MPa, respectively. The bending strengths of TLF/PHBV with different LF contents were greater than those of ULF/PHBV composites (Table 3). This could be due to the fact that most of the impurities such as pectin, lignin, and hemicellulose in LF were dissolved and removed after treatment with NaOH–H_2_O_2_, making the surface of the fiber rougher. The bonding area and mechanical bonding forces between LF and PHBV increased, due to which the bending property was improved [42].

#### 3.2.5. Mechanical Properties after Soaking

**Figure 10:**
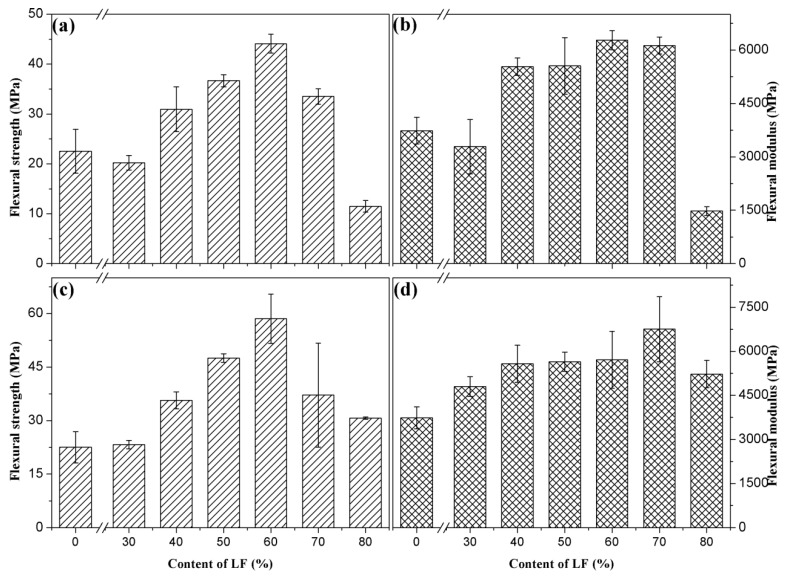
Flexural strengths and moduli of composites after immersion in water: (**a**,**b**) ULF/PHBV and (**c**,**d**) TLF/PHBV.

The prepared specimens were soaked in water at room temperature for 24 h, after which they were taken out and their bending properties were tested. Figure 10 shows the changes in flexural strengths and moduli of ULF/PHBV and TLF/PHBV composites after immersion in water. The trends of flexural strengths and moduli of composites after immersion were consistent with those before immersion. This indicated that with an increase in LF content, the flexural strength increased at first and then decreased. Compared to the flexural strength before immersion (Table 3), the flexural strengths of ULF/PHBV and TLF/PHBV decreased to varying extents after immersion. However, the flexural strengths of TLF/PHBV composites were still higher than those of ULF/PHBV. When LF content was 60%, the flexural strengths of ULF/PHBV and TLF/PHBV were the highest and their values reached 44.12 MPa and 58.55 MPa, respectively. This was a decrease of 70.5% and 55.0%, respectively, compared to the corresponding composites before immersion. This could be due to an increase in volume of LF after water absorption and adhesion to PHBV were reduced. The compatibility at the surface and the interfacial affinity decreased, which in turn decreased the bending strength of the composite [43]. On the other hand, a decrease in bending strengths of TLF/PHBV composites was less compared to ULF/PHBV composites, which indicated that the bonding between LF and PHBV was tighter and the interfacial properties of composites were improved after NaOH–H_2_O_2_ treatment of LF. The void ratio in the composite was significantly reduced and the moisture could hardly penetrate, which greatly reduced the damage to the composite from immersion in water [44].

#### 3.2.6. SEM

**Figure 11:**
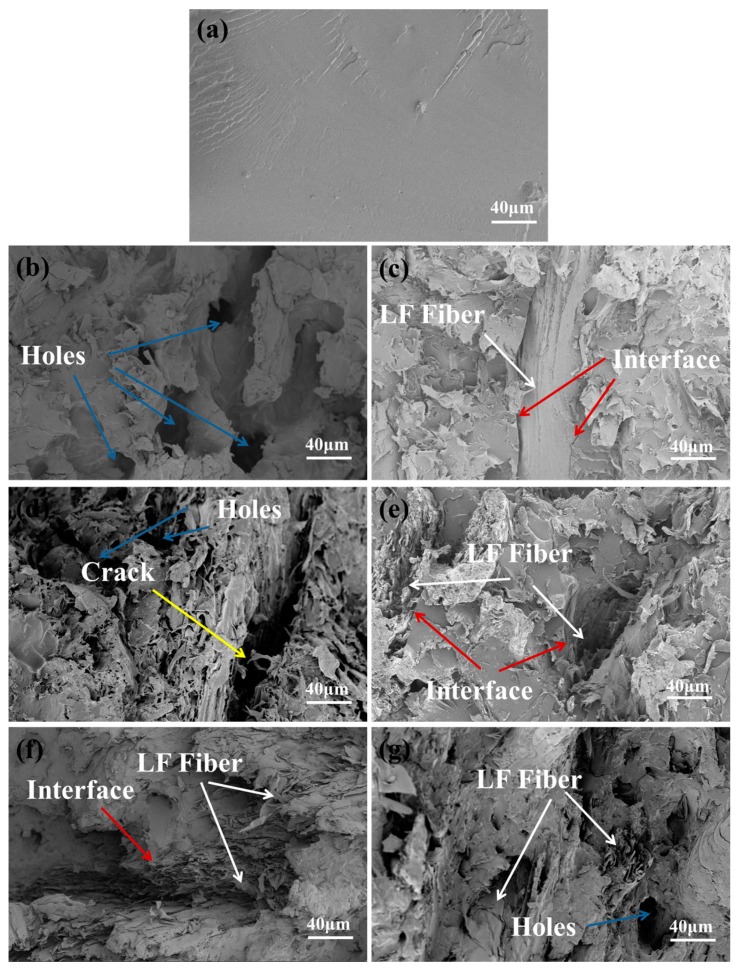
SEM images of composites: (**a**) PHBV, (**b**) 30% ULF/PHBV, (**c**) 60% ULF/PHBV, (**d**) 80% ULF/PHBV, (**e**) 30% TLF/PHBV, (**f**) 60% TLF/PHBV, and (**g**) 80% TLF/PHBV.

Figure 11 shows the SEM images of the bending section of PHBV, ULF/PHBV, and TLF/PHBV composites. The fractured surface of PHBV is shown in Figure 11a, which indicated that the fracture mode of pure PHBV was brittle fracture. Figure 11b–d show the bent sections of ULF/PHBV composites with LF contents of 30%, 60%, and 80%, respectively. In Figure 11b, it was obvious that holes were left in the section of the fiber. This indicated that when the LF content was low, the bending load was not carried by the fibers, but by PHBV. The addition of fibers destroyed the continuity of the matrix, which negatively affected the mechanical properties. When the fiber content reached 60% (Figure 11c), LF was sufficiently wrapped by PHBV. Hence, it could uniformly disperse the load and withstand it, thereby increasing the mechanical properties of the composite. However, LF and PHBV were poorly combined at the interface, which limited the mechanical properties of the composites. When the LF content was 80% (Figure 11d), a large number of fibers were accumulated and were exposed to the outer-side of the substrate. Large cracks developed between the fibers, which significantly reduced the mechanical properties of the composite. Figure 11e–g show the curved sections of TLF/PHBV composites with LF contents of 30%, 60%, and 80%. Compared with ULF/PHBV, the interfacial bonding performance was significantly improved and the LF was wrapped by the PHBV matrix. There was no fiber pull-out and fracture and hence the fiber was more tightly bonded to the matrix.

**Figure 12:**
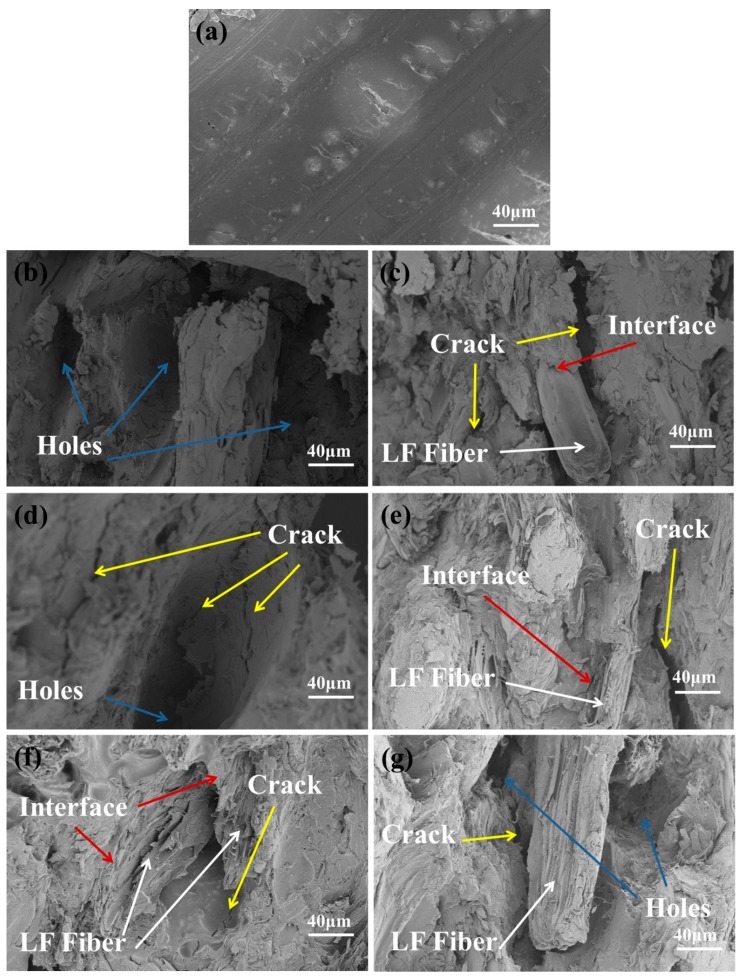
SEM images of the composites after immersion: (**a**) PHBV, (**b**) 30% ULF/PHBV, (**c**) 60% ULF/PHBV, (**d**) 80% ULF/PHBV, (**e**) 30% TLF/PHBV, (**f**) 60% TLF/PHBV, and (**g**) 80% TLF/PHBV.

Figure 12 shows the images of curved sections of composites after immersion in water. Figure 12a shows the fractured surface of pure PHBV after immersion in water, which remained substantially unchanged and still displayed typical brittle fracture. Figure 12b–d show the curved sections of ULF/PHBV composite, and Figure 12e–g show the curved sections of TLF/PHBV composite with LF contents of 30%, 60%, and 80%, respectively, after immersion in water. It was observed that after ULF/PHBV was immersed in water, the fibers were pulled out and completely debonded from PHBV, due to which the PHBV matrix produced many cracks of different sizes. After TLF/PHBV was immersed in water, interfacial debonding also occurred between the matrix and fibers. However, LF was not pulled out and only a few microcracks were generated within the PHBV matrix. Similar to the results of M. Su et al., LFs increased the flexural strength and flexural modulus of PHBV and prolonged the time of thermal decomposition [45].

After water absorption the compatibility between the fiber and PHBV was lowered, the fiber was peeled off, and the interfacial adhesion was weakened. In addition, when the swelling of the fibers generated stress at the interface, microcracks were generated in the matrix surrounding the swollen fibers. Therefore, the bending properties of the composite were lowered. The interfacial compatibility and moisture resistance of LF and PHBV after NaOH–H_2_O_2_ treatment were enhanced. Therefore, the bending ability of the composite after immersion was reduced to a lesser extent.

## 4. Conclusions

LFs and LFs treated with NaOH–H_2_O_2_ were used for preparing composites to enhance the properties of PHBV. The effects of modification on the properties of LF and its composites were studied. The results of this work were as follows:

1. NaOH–H_2_O_2_ treatment removed impurities such as pectin and nitrogen containing groups on the surface of LF. Hemicellulose and lignin were also effectively removed. At the same time, the moisture absorption rate of the fibers was reduced and the surface roughness of LF was improved. Thus, its combination with PHBV was facilitated.

2. On addition of different amounts of LF, the water absorption rates of the composites increased continuously. Their bending strength increased at first and then decreased. Composite with 60% LF content had the highest bending strength. Compared with ULF/PHBV, the water absorption rate of TLF/PHBV decreased, whereas its bending strength increased to some extent. This was mainly because NaOH–H_2_O_2_ treatment removed hemicellulose and lignin. The number and types of surface hydroxyl groups of the fiber were reduced, and hence the contact area and interfacial bonding between the fiber and matrix were greatly improved. The moisture resistance of the composite material was further improved, so that the loss of bending property after immersion of TLF/PHBV in water was less than that of ULF/PHBV.

3. SEM of the curved section of the composite further confirmed that the fibers in TLF/PHBV were more tightly bound to PHBV than in ULF/PHBV, showing good interfacial adhesion. This good interfacial compatibility was retained even after immersion in water.

## Figures and Tables

**Table 1:** Chemical composition of luffa fibers (LFs) before and after treatment [29].

Sample	*α*-Cellulose (%)	Hemicellulose (%)	Lignin (%)	Moisture Regained (%)
ULF	57.51 ± 0.20	29.47 ± 0.25	20.45 ± 0.29	8.83 ± 0.3
TLF	68.29 ± 0.23	14.62 ± 0.14	16.91 ± 0.48	6.27 ± 0.4

**Table 2:** TG and DTG data of composites.

Sample	LF Content	*T*_initial_ (°C)	Peak Degradation Temperature (°C)	Residual Mass Fraction (%)
PHBV	0%	313.9	349.4	0
ULF/PHBV	30%	298.4	325.4	3.45
	40%	303.9	343.3	4.13
	50%	304.9	346.5	2.70
	60%	296.0	338.2	7.35
	70%	282.9	335.1	8.81
	80%	301.5	346.3	9.51
TLF/PHBV	30%	291.2	320.9	0.38
	40%	292.7	316.8	3.92
	50%	290.6	314.9	5.32
	60%	300.8	347.5	10.91
	70%	288.1	337.8	6.98
	80%	294.5	337.4	8.17

**Table 3:** Flexural strengths of composites before and after immersion in water.

Strength (MPa)	0%	30%	40%	50%	60%	70%	80%
ULF/PHBV	23.53	37.31	56.56	63.87	75.23	72.84	38.87
TLF/PHBV	23.53	42.55	64.26	81.77	90.73	79.50	72.84
ULF/PHBV after immersion	22.52	20.22	30.97	36.67	44.12	33.52	11.5
TLF/PHBV after immersion	22.52	23.29	35.65	47.50	58.55	37.12	30.69
